# Emerging Human Resource Trends in Academic Libraries

**DOI:** 10.5195/jmla.2021.1320

**Published:** 2021-10-01

**Authors:** Tamara M. Nelson

**Affiliations:** 1 tnelso24@uthsc.edu, University of Tennessee Health Science Center, Memphis, TN

*Emerging Human Resource Trends in Academic Libraries* is a compilation of current human resource issues often faced in academic librarianship. Topics are presented using a research-based approach aimed at providing readers with relevant accounts and practical solutions from contributors representing diverse backgrounds. The book also discusses the lack of preparation in library science programs to prepare graduates for issues they may face upon entering the workforce. It is a useful resource for academic librarians or those associated with human resources in an academic library setting, as well as students and other individuals preparing to enter the field.

The book begins with an introduction written by the editors and is then organized into five distinct sections centered around trending topics. Part I, “Academic Environment and Library Organizations,” presents the current state of the academic library culture and human resource challenges that are being faced. The chapters in this section cover an overview of a major undertaking by one institution to completely overhaul their human resource model, recruitment of subject matter experts, and faculty status.

Part II, entitled “Education of the Professional Librarian,” tackles the trending topic of whether library science (LIS) education properly prepares students to be academic librarians but also presents topics not directly relating to the education of a professional librarian that are equally important and trending in the profession. This section opens with a chapter dedicated to diversity, equity, and inclusion (DEI) in academic libraries. DEI is an important topic that has garnered more attention amidst a current rise in issues of systemic racism in America. The connection between this topic and education of the professional librarian is a stretch, but the authors of the chapter provide an effective blueprint for incorporating DEI in the academic library setting. Considering the complexity of DEI, it could have been an entire section. In the next chapter, the author discusses the responsiveness of LIS graduate school programs to the emerging roles of academic librarians. Lastly, this section ends with a discussion of the skills needed by academic librarians to work with various stakeholders.

Part III, “Professional Development,” is dedicated to emerging human resource trends in how academic libraries provide the means for professional growth among employees. Chapter 7 discusses employee engagement and provides examples of how libraries can proactively build a plan to keep employees motivated and engaged. The next chapter outlines the success of one library's implementation of a career development program and how it impacted recruitment and retention. In the final chapter of this section, the author discusses developing a mentorship program and peer coaching to support professional growth within the profession.

“Leadership in Practice” is the title of part IV and focuses on how leaders in academic libraries can advocate and make a difference at their institution. In chapter 10, the author discusses how human resource department leadership can demonstrate the library's value through better communication, leadership training, and other means. Chapter 11 focuses on the ever-trending idea of librarians as change agents. The author presents many models on navigating institutional change. Lastly, this section ends with a chapter dedicated to developing a community of practice specially for midlevel managers.

Part V, the final part of the book, discusses other trends in the profession related to staff and paraprofessional roles. For example, technical services have gone through many drastic changes, and chapter 13 offers the answers to human resource questions that may arise as academic libraries deal with these changes. Chapter 14 presents ways to make staff and faculty feel equally valued. The book concludes with chapter 15, a discussion on ways to professionally grow and engage library workers who do not hold a library science degree.

*Emerging Human Resource Trends in Academic Libraries* is a strong attempt to present high-ranking human resource concerns of the profession today. It offers practical advice and resources for further exploration of topics. Academic librarians, human resource professionals, and students would find the information provided useful.

